# KPNA2 expression is a potential marker for differential diagnosis between osteosarcomas and other malignant bone tumor mimics

**DOI:** 10.1186/s13000-020-01051-6

**Published:** 2020-11-11

**Authors:** Lucen Jiang, Jianghuan Liu, Qingzhu Wei, Yiyang Wang

**Affiliations:** 1 Department of Pathology, The Third Affiliated Hospital of Southern Medical University, Guangzhou, 510630 Guangdong China; 2 Department of Pathophysiology, School of Medicine, Jinan University, Guangzhou, 510632 Guangdong China

**Keywords:** Osteosarcomas, Karyopherin α2 (KPNA2), Diagnosis, Biomarker

## Abstract

**Background:**

Karyopherin α2 (KPNA2), a member of the karyopherin α family, has been studied in several cancers but has not yet been substantially investigated in malignant bone tumors. The purpose of the current study was to evaluate the KPNA2 expression level and its utility as a novel diagnostic biomarker in osteosarcomas and malignant bone tumor mimics, such as chondrosarcomas and Ewing sarcomas (ESs).

**Method:**

We investigated the expression of KPNA2 protein by immunohistochemistry on paraffin-embedded surgical specimens from 223 patients with malignant and benign bone tumors, including 81 osteosarcomas, 42 chondrosarcomas, 15 ESs, 28 osteoid osteomas, 20 osteochondromas and 37 chondroblastomas. Immunoreactivity was scored semiquantitatively based on staining extent and intensity.

**Results:**

Sixty-seven of 81 (82.7%) osteosarcoma, zero of 42 (0%) chondrosarcoma and one of 15 (6.7%) ES samples showed immunoreactivity for KPNA2. Negative KPNA2 expression was observed in all benign bone tumors. The expression of KPNA2 in osteosarcoma samples was much higher than that in chondrosarcoma and ES samples (*P* < 0.001). The sensitivity and specificity of KPNA2 immunoexpression for detecting osteosarcoma were 82.7 and 100%, respectively. Several subtypes of osteosarcoma were analyzed, and immunostaining of KPNA2 was frequent in osteoblastic samples (90.9%), with 39 samples (70.9%) showing strong-intensity staining. KPNA2 positivity was observed in ten of 13 (76.9%) chondroblastic, two of 6 (33.3%) fibroblastic, three of 4 (75%) telangiectatic and two of 3 (66.7%) giant cell-rich osteosarcoma samples. The strongest intensity staining was observed in osteoblastic osteosarcoma.

**Conclusion:**

KPNA2 is frequently expressed in osteosarcomas, particularly in osteoblastic and chondroblastic tumors, but is rarely positive in chondrosarcomas and ESs. This feature may aid in distinguishing between osteosarcoma and other bone sarcoma mimics. This report supports KPNA2 as a novel marker for the diagnosis of osteosarcoma.

## Background

Osteosarcoma is defined by the presence of malignant cells producing osteoid or immature bone. It represents the most common primary skeletal sarcomas and has a high prevalence in children, adolescents and young adults [1]. Surgery combined with neoadjuvant chemotherapy is the main therapeutic strategy for the treatment of osteosarcoma patients, and the five-year survival rate has markedly improved to over 60% in patients with localized tumors [2]. Accurate distinction between osteosarcoma and other sarcomas of bone is very important, as chemotherapeutic and surgical approaches differ significantly between these tumor types and are dependent mainly on the histopathological diagnosis [3].

The identification of an osteoid matrix is necessary for osteosarcoma diagnosis. However, this diagnostic feature may be difficult to detect in many cases, and the diagnosis becomes challenging when minimal or scant osteoid matrix formation is identified on biopsy. Immunohistochemistry has been evaluated as an adjunct to offer additional information to support the final diagnosis [4]. Early investigations suggested that osteonectin was a sensitive and specific marker of osteoblastic differentiation that could be helpful in the diagnosis of bone tumors, particularly osteosarcomas [5]. Subsequent studies, however, have demonstrated that osteonectin can be detected in other primary bone sarcomas, including Ewing sarcoma (ES) and chondrosarcoma [6]. Some proteins have been identified, such as DMP-1, CADM1, galectin-1 and NDRG1, as potential markers of osteosarcoma, but their practical utility in the diagnosis of bone tumors is unclear [7–10]. SATB2 is known to play a role in the regulation of osteoblast differentiation, and the SATB2 level measured via immunohistochemistry has shown promise as a highly sensitive marker of osteoblasts [11]. However, SATB2 positivity is not specific for osteosarcoma and cannot differentiate it from other primary bone sarcomas, which has been well documented by Davis and Horvai [12]. Therefore, highly sensitive and specific biomarkers for osteosarcoma differential diagnosis urgently need to be identified.

Karyopherin α2 (KPNA2), an adaptor protein, is a member of the karyopherin α protein family that plays a crucial role in the transportation of proteins from the cytoplasm into the nucleus [13]. Together with importin-β, KPNA2 mediates the nuclear translocation of numerous target proteins through the nuclear pore complex via recognition of nuclear localization signals [14]. Previous studies have demonstrated that KPNA2 is a potential biomarker in multiple forms of cancer, including breast cancer, lung cancer, gastric cancer, colon cancer, prostate cancer and upper tract urothelial carcinoma [15–18]. The expression of KPNA2 has been reported to be associated with poor prognosis in patients with esophageal squamous cell carcinoma, epithelial ovarian carcinoma and small hepatocellular carcinoma [19–21]. However, in human malignant bone tumors, the expression level of KPNA2 has not been clarified.

The purpose of the present study was to evaluate the expression of KPNA2 in osteosarcoma, chondrosarcoma and ES samples using immunohistochemistry to confirm its potential diagnostic utility as a novel molecular marker for discriminating osteosarcoma from other primary bone sarcomas.

## Methods

### Tissue samples

This study was approved by the relevant institutional review board. In total, whole tissue sections of 138 malignant and 85 benign bone tumors were evaluated for expression of KPNA2: 81 osteosarcomas (55 osteoblastic, 13 chondroblastic, 6 fibroblastic, 4 telangiectatic and 3 giant cell-rich osteosarcomas), 42 chondrosarcomas (26 grade I, 9 grade II, and 7 grade III chondrosarcomas), 15 ESs, 20 osteoid osteomas, 28 osteochondromas and 37 chondroblastomas. All tissue samples accessioned between January 2014 and September 2020 were retrieved from the surgical pathology and consultation files of the Third Affiliated Hospital of Southern Medical University. The diagnosis of malignant and benign bone tumors was established based on tumor location, histomorphology, and/or the results of immunohistochemical studies. All biopsy slides were examined independently by two experienced pathologists (Lucen Jiang and Qingzhu Wei). The histopathological diagnosis of each biopsy was made according to WHO classification. The clinicopathological features of patients with malignant bone tumors are presented in Table 1.

**Table 1:** Clinicopathological features of osteosarcoma, chondrosarcomas and Ewing sarcoma

	Total	Osteosarcoma	chondrosarcomas	Ewing sarcoma
**Number of patients**	138	81	42	15
**Gender**				
Male, n (%)	78 (56.5)	46 (56.8)	21 (50)	11 (73.3)
Female, n (%)	60 (43.5)	35 (43.2)	21 (50)	4 (26.6)
**Age, (Years)**				
Range	1–69	5–66	3–69	1–27
Median	24	23	34	16
**Distribution, n (%)**				
Femur	61 (44.2)	43 (53.1)	14 (33.3)	4 (26.7)
Tibia/Fibula	27 (19.6)	16 (19.8)	7 (16.7)	4 (26.7)
Pelvis	17 (12.3)	7 (8.6)	6 (14.2)	4 (26.7)
Humerus	11 (8.0)	6 (7.4)	5 (11.9)	0 (0)
Spine/Sacrum	12 (8.7)	5 (6.2)	5 (11.9)	2 (13.3)
Astragalus	3 (2.2)	2 (2.5)	1 (2.4)	0 (0)
Ribs	2 (1.4)	1 (1.2)	1 (2.4)	0 (0)
Facial bones	2 (1.4)	1 (1.2)	1 (2.4)	0 (0)
Scapula	3 (2.2)	0 (0)	2 (4.8)	1 (6.7)

### Immunohistochemical analysis

All bone tissue samples were fixed in formalin (pH 7.4) and decalcified using standard procedures with 16–20 h incubation in 5% aqueous hydrochloric acid (HCl). Immunohistochemistry was performed on 4-μm-thick paraffin-embedded whole tissue sections with standard techniques. Briefly, following deparaffinization and rehydration, charged slides with 4-μm-thick sections of tissue were treated with 3% hydrogen peroxide to eliminate endogenous peroxidase activity and then processed for antigen retrieval with 10 mm citrate buffer (pH 6.0) for 15 min at 95 °C, followed by incubation in 5% bovine serum albumin (BSA) for 20 min at room temperature. All sections were rinsed with phosphate-buffered saline (PBS) and incubated overnight at 4 °C with a monoclonal mouse anti-human antibody against KPNA2 (sc-55,538, 1:100 dilution, Santa Cruz Biotechnology, Santa Cruz, USA). The slides were washed with PBS and then incubated at room temperature with the appropriate secondary antibody for 20 min. Diaminobenzidine (DAB) staining was then performed. Cervical cancer samples were used as positive controls. Negative control sections were prepared by substituting a nonimmune IgG antibody for the primary antibody.

### Analysis of immunohistochemical staining

The percentage of KPNA2-positive cells and the staining intensity were scored in a semiquantitative manner. Immunohistochemical slides were scanned and evaluated by two experienced researchers (Lucen Jiang and Qingzhu Wei) who were blinded to the clinical data of the patients. The proportion of cells with nuclear KPNA2 staining was scored as follows: 0, no staining; 1+, < 5% of cells with positive staining; 2+, 5–25% of cells with positive staining; 3+, 26–50% of cells with positive staining; 4+, 51–75% of cells with positive staining; and 5+, 76–100% of cells with positive staining. Staining intensity was also graded as weak, moderate, or strong. KPNA2 nuclear expression was classified as positive if it was observed in at least 5% of the cancer cells; if it was present in less than 5% of cells, the sample was classified as having negative expression.

### Statistical analysis

Comparisons of KPNA2-positive expression between osteosarcoma and chondrosarcoma and ES samples were performed using the χ^2^ test followed by the Pearson chi-squared test. Differences were considered statistically significant at the level of *P* < 0.05. SPSS software was used to analyze the data.

## Results

### Patient characteristics

The clinicopathological characteristics of patients with malignant bone tumors are shown in Table 1. The data for 81 osteosarcoma (46 males and 35 females), 42 chondrosarcoma (21 males and 21 females) and 15 ES (11 males and 4 females) patients with both clinical and KPNA2 expression data were retrieved from hospital data files in 2020. The median ages at the time of diagnosis were 23 years (range 5–66 years), 34 years (range 3–69 years), and 16 years (range 1–27 years) in osteosarcoma, chondrosarcomas and ES patients, respectively. The distribution of tumor sites mainly included the femur, tibia/fibula and pelvis. The locations of the three primary bone sarcomas were similar. Of all 137 patients, most patients (44.2%) had tumors in the femur, 19.6% of patients had tumors in the tibia/fibula, and 12.3% of patients had tumors in the pelvis.

### Immunohistochemical analysis of KPNA2 in osteosarcoma, chondrosarcoma, ES and benign bone tumor tissues

**Fig. 1:**
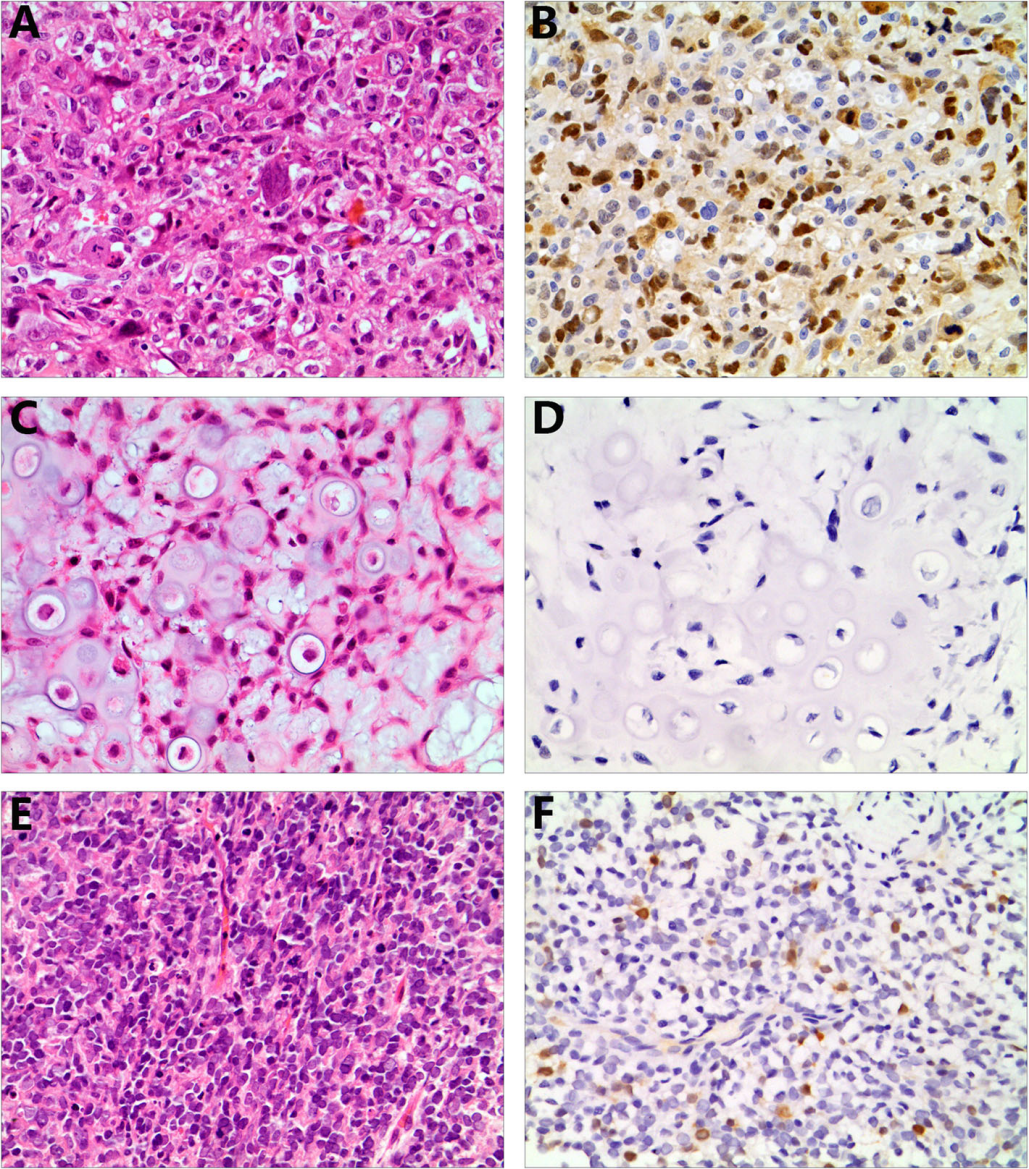
KPNA2 immunostaining in osteosarcoma, chondrosarcoma and Ewing sarcoma samples. Osteosarcoma samples (**a**) showed strong staining for KPNA2 (**b**). Chondrosarcoma (**c**) and Ewing sarcoma (**e**) samples were negative for KPNA2 (**d** and **f**). (**a**–**f** × 200)

**Fig. 2:**
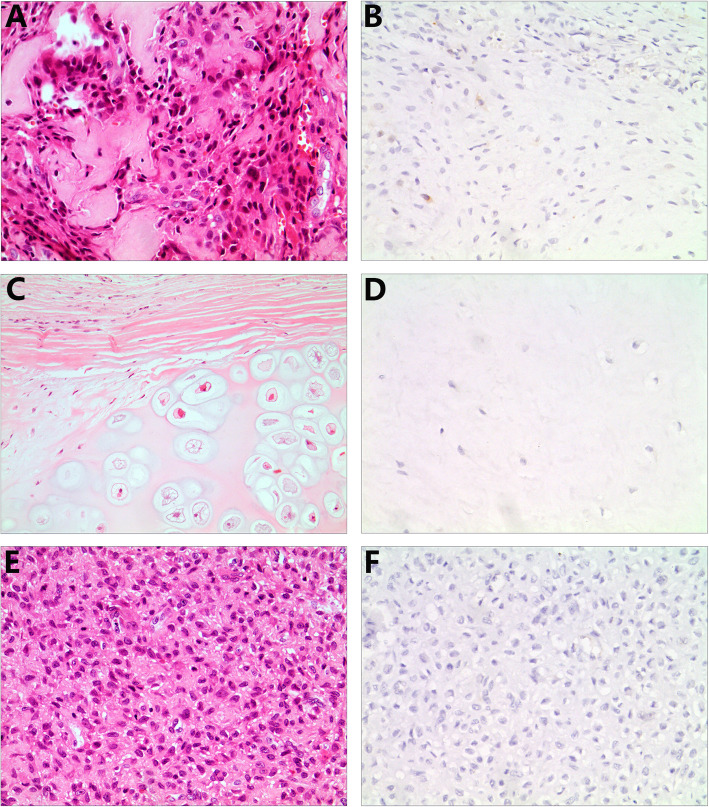
KPNA2 expression in benign bone tumors. Osteoid osteoma (**a**), osteochondroma (**c**) and chondroblastoma (**e**) samples were negative for KPNA2 (**b**, **d** and **f**). (**a–f** × 200)

The immunohistochemical findings in malignant and benign bone tumors are summarized in Table 2. Of the osteosarcoma samples, 67 of 81 (82.7%) were positive for KPNA2. KPNA2 expression was predominantly observed in the nuclei of tumor cells, with little expression observed in the cytoplasm (Fig. 1 a & b). To evaluate the utility of KPNA2 in distinguishing tumors of osteoblastic origin from histological mimics, we examined chondrosarcoma and ES samples. As shown in Table 2, KPNA2 was expressed in a lower proportion of chondrosarcoma (0) and ES (6.7%) samples than osteosarcoma samples, and weak as well as diffuse staining was detected only in tumors (Fig. 1 c–f). All samples from benign bone tumors including osteoid osteomas, osteochondromas and chondroblastomas were negative for KPNA2 (Table 2; Fig. 2 a-f). Therefore, the sensitivity and specificity of KPNA2 immunoexpression were 82.7 and 100%, respectively, in osteosarcoma samples. Therefore, the proportion and intensity of KPNA2 expression found in osteosarcomas were obviously stronger than the respective levels in chondrosarcoma and ES samples (*P* < 0.001).

**Table 2:** Summary of immunohistochemical staining for KPNA2 in bone tumors

Bone tumors	No. of cases	No. (%) of positive cases	No. (%) of negative cases
**Malignant bone tumors**			
Osteosarcomas	81	67 (82.7)	14 (17.3)
Chondrosarcomas	42	0 (0)	42 (100)
Ewing sarcoma	15	1 (6.7)	14 (93.3)
**Benign bone tumors**			
Osteoid osteoma	28	0 (0)	28 (100)
Osteochondroma	20	0 (0)	28 (100)
Chondroblastoma	37	0 (0)	37 (100)

### Evaluation of KPNA2 staining extent and intensity in osteosarcoma subtypes

**Fig. 3:**
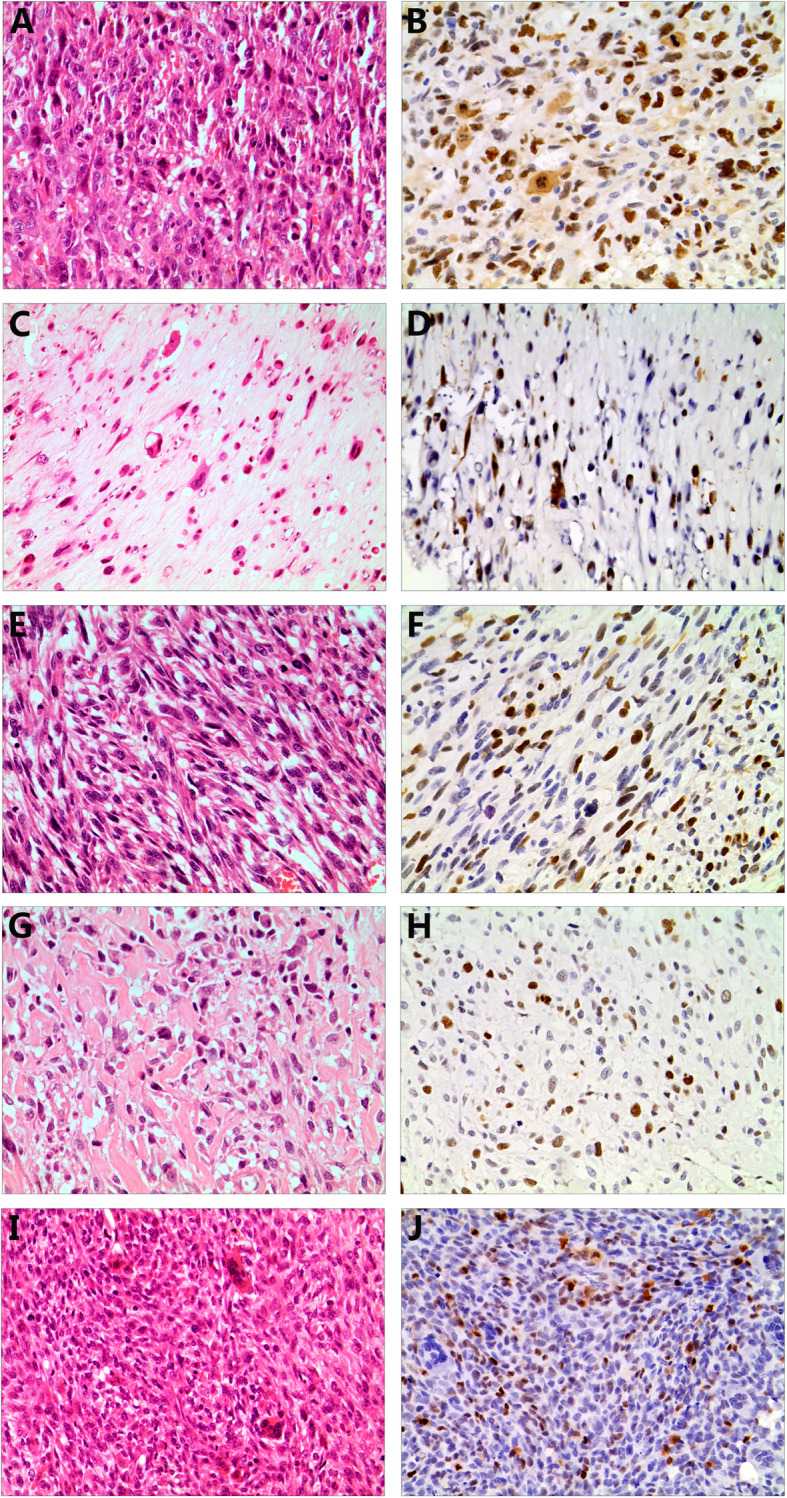
KPNA2 expression in subtypes of osteosarcoma. Osteoblastic (**a**), chondroblastic (**c**), fibroblastic (**e**), telangiectatic (**g**) and giant cell-rich osteosarcoma (**i**) samples were positive for KPNA2. (**a–j** × 200)

Three major subtypes of conventional osteosarcoma are recognized, reflecting the predominant form of the tumor matrix: the osteoblastic, chondroblastic and fibroblastic subtypes. We detected expression of KPNA2 via immunohistochemical staining in the different osteosarcoma types, including three conventional and two other subtypes. The 81 osteosarcomas consisted of 55 osteoblastic, 13 chondroblastic, 6 fibroblastic, 4 telangiectatic and 3 giant cell-rich osteosarcoma surgical resection specimens, all from unique patients. The KPNA2 immunohistochemistry results for the osteosarcoma subtypes are summarized in Table 3. Of the osteoblastic samples, fifty of 55 (90.9%) were positive for KPNA2, with 39 (70.9%) samples showing strong-intensity staining (Fig. 3 a & b). KPNA2 was expressed in ten of 13 (76.9%) chondroblastic samples, with 7 (53.8%) samples showing strong-intensity staining (Fig. 3 c & d). Positive KPNA2 expression was observed in two of 6 (33.3%) fibroblastic osteosarcomas, with 2 (33.3%) samples showing strong-intensity staining (Fig. 3 e & f). Three of 4 (75%) telangiectatic and two of 3 (66.7%) giant cell-rich osteosarcoma samples had positive KPNA2 staining, with 50 and 33.3% of samples showing strong-intensity staining, respectively (Fig. 3 g-j). When KPNA2 expression was evaluated separately in the various subtypes of conventional osteosarcoma samples, osteoblastic and chondroblastic samples showed the greatest extent of staining, whereas more limited staining was observed in the fibroblastic samples. Telangiectatic and giant cell-rich osteosarcoma samples were also reactive for KPNA2, but the pattern of staining was generally not as extensive as that in osteoblastic osteosarcomas. Overall, both the extent and intensity of KPNA2 immunoexpression were highest in osteoblastic osteosarcomas among the types of bone tumors analyzed.

**Table 3:** Expression and extent of KPNA2 Immunohistochemical staining in osteosarcoma subtypes

Osteosarcoma subtype	Positives Cases^b^ (%)	Extent of staining^a^						Strong-intensity staining (%)
		0	1+	2+	3+	4+	5+	
Osteoblastic (*n* = 55)	50 (90.9)	3	2	13	18	15	4	39 (70.9)
Chondroblastic (*n* = 13)	10 (76.9)	2	1	3	4	2	1	7 (53.8)
Fibroblastic (*n* = 6)	2 (33.3)	3	1	0	2	0	0	2 (33.3)
Telangiectatic (*n* = 4)	3 (75)	1	0	1	2	0	0	2 (50)
giant cell-rich (*n* = 3)	2 (66.7)	1	0	1	1	0	0	1 (33.3)
Total (*n* = 81)	67 (82.7)	10	4	18	27	17	5	51 (63.0)

^a^0, no staining; 1+, < 5%; 2+, 5–25%; 3+, 26–50%; 4+, 51–75%;5+, 76–100%

^b^The positive KPNA2 staining was defined as nucleus staining in at least 5% cells

## Discussion

In this study, we carried out a comparative analysis of KPNA2 expression profiles in osteosarcoma, chondrosarcoma, ES and several benign bone tumor clinical samples. The tissues of all bone tumors were decalcified in 5% HCl within 24 h to avoid lessening the reactivity of diagnostically useful antigens [22]. The decalcification protocol remained the same throughout the entire case collection period. We discovered that KPNA2 expression was significantly higher in osteosarcoma patient samples than in samples of other bone tumors. As such, KPNA2 can serve as a novel biomarker for diagnosing osteosarcoma. In addition, we observed that the proportion of cells with KPNA2-positive staining was markedly higher and the intensity of KPNA2-positive staining was obviously stronger in osteoblastic cases than in other subtypes.

Osteosarcoma is the most common primary malignant bone tumor in children, adolescents, and young adults. In cancer registry data with histological stratification, osteosarcoma cases account for approximately 35% of cases, followed by chondrosarcoma (25%) and ES (16%) cases. Accurate diagnosis of each type of sarcoma is critical for ensuring appropriate therapy for patients. Immunohistochemistry is very important for confirming a diagnosis of osteosarcoma over other high-grade bone sarcomas when bone or osteoid production is not overtly apparent on biopsy. Some osteosarcomas can be accurately classified based on expression of one particular sensitive biomarker, SATB2. As a nuclear antigen directly involved in osteoblast lineage commitment, SATB2 is an attractive target for immunohistochemical identification of osteoblasts. However, the results obtained by Davis and Horvai suggested that SATB2 positivity is not specific for osteosarcoma compared with other primary bone sarcomas [12]. Therefore, an additional biomarker to assist in accurately stratifying patients into the correct diagnostic category of bone sarcoma would be beneficial.

In recent years, KPNA2 has emerged as a potential biomarker in multiple solid tumor types, and its aberrant expression is often associated with poor prognosis in patients. There was a trend toward a lower degree of differentiation and higher pathological state in the high KPNA2 expression group compared with the low KPNA2 expression group. Alshareeda et al. found that KPNA2 significantly contributed to aberrant localization of key proteins and predicted poor prognosis in breast cancer [15]. Altan et al. proved that KPNA2 expression in primary lesions and metastatic lymph nodes was associated with poor prognosis and progression in gastric cancer [17]. Zhang et al. reported that KPNA2 was a novel prognostic marker and a potential therapeutic target for colon cancer [18]. In our study, we analyzed KPNA2 expression in osteosarcoma versus chondrosarcoma and ES samples and observed KPNA2 immunoreactivity in 81 osteosarcoma, 42 chondrosarcoma and 15 ES sections. The results of the current study indicate that the expression of KPNA2 in osteosarcoma samples is much higher than that in ES samples; in addition, KPNA2 expression was not observed in chondrosarcoma and benign bone tumor samples. Our research reveals that KPNA2 expression is a sensitive and specific marker for osteosarcoma compared with chondrosarcoma and ES. This is the first report of a correlation between KPNA2 expression and sarcoma. The results of the present study show that the expression of KPNA2 was significantly higher in osteosarcoma than benign bone tumor tissues. Our data demonstrate that KPNA2 is sufficiently reliable for use as a diagnostic marker in osteosarcoma biopsy samples.

We did note that the proportion of cells with positive KPNA2 expression was higher and the intensity of KPNA2 immunostaining was stronger in osteosarcoma samples than in samples of other high-grade bone sarcomas. However, variations in the extent and intensity of staining were observed within osteosarcoma subtypes, ranging from weak to diffuse to strong nuclear reactivity. Notably, KPNA2 expression was observed in a higher proportion of osteoblastic samples than samples of the other subtypes, and the osteoblastic samples generally showed stronger staining than samples of the other subtypes. In chondroblastic tissue, all but three samples expressed KPNA2 (76.9%), with 53.8% of samples showing strong-intensity staining, second to only osteoblastic samples. Moreover, despite there being fewer than six fibroblastic, telangiectatic and giant cell-rich osteosarcoma samples, the tendency for positive KPNA2 expression in these subtypes agrees with the results seen in osteoblastic and chondroblastic samples. Our results are promising, and further studies with larger cohorts and diverse sample types to validate the findings are warranted. All samples of osteoid osteoma, osteochondroma and chondroblastoma had negative KPNA2 staining. This finding is consistent with those of previous studies demonstrating that benign tissues are negative for KPNA2 expression [23].

## Conclusion

In summary, we found for the first time that KPNA2 immunohistochemical expression was highly sensitive and specific for osteosarcoma compared with chondrosarcoma and ES. This study reveals that KPNA2 immunoexpression may be a potential marker for differentiating osteosarcoma, particularly the osteoblastic and chondroblastic subtypes, from chondrosarcoma and ES. Although the identification of malignant osteoid matrix and the combined consideration of clinical and radiological data remain cornerstones of osteosarcoma diagnosis, our results strongly support that KPNA2 expression can serve as an additional diagnostic marker to improve the diagnosis of osteosarcoma.

## Data Availability

All data generated or analyzed during this study are included in this published article. The data that support the findings of this study are available.

## Abbreviations

KPNA2: Karyopherin α 2
ES: Ewing sarcomas (ES)
